# GOLIAH (Gaming Open Library for Intervention in Autism at Home): a 6-month single blind matched controlled exploratory study

**DOI:** 10.1186/s13034-017-0154-7

**Published:** 2017-03-22

**Authors:** Anne-Lise Jouen, Antonio Narzisi, Jean Xavier, Elodie Tilmont, Nicolas Bodeau, Valentina Bono, Nabila Ketem-Premel, Salvatore Anzalone, Koushik Maharatna, Mohamed Chetouani, Filippo Muratori, David Cohen, the MICHELANGELO Study Group, Silvio Bonfiglio, Fabio Apicella, Federico Sicca, Lucia Billeci, Giovanni Pioggia, Federico Cruciani, Cristiano Paggetti, Angele Giuliano, Maryrose Francisa, Saptarshi Das, Wasifa Jamal, Leo Galway, Mark Donnelly

**Affiliations:** 1 Institute of Intelligent Systems and Robotics, University Pierre and Marie Curie, 75005 Paris, France; 2 Department of Developmental Neuroscience, IRCCS Stella Maris Foundation, Viale del Tirreno, 331, 56018 Calambrone, Pisa, Italy; 3 Department of Child and Adolescent Psychiatry, APHP, Groupe Hospitalier Pitié-Salpêtrière et University Pierre and Marie Curie, 75013 Paris, France; 4 School of Electronics and Computer Science, University of Southampton, Southampton, SO17 1BJ UK

## Abstract

**Background:**

To meet the required hours of intensive intervention for treating children with autism spectrum disorder (ASD), we developed an automated serious gaming platform (11 games) to deliver intervention at home (GOLIAH) by mapping the imitation and joint attention (JA) subset of age-adapted stimuli from the Early Start Denver Model (ESDM) intervention. Here, we report the results of a 6-month matched controlled exploratory study.

**Methods:**

From two specialized clinics, we included 14 children (age range 5–8 years) with ASD and 10 controls matched for gender, age, sites, and treatment as usual (TAU). Participants from the experimental group received in addition to TAU four 30-min sessions with GOLIAH per week at home and one at hospital for 6 months. Statistics were performed using Linear Mixed Models.

**Results:**

Children and parents participated in 40% of the planned sessions. They were able to use the 11 games, and participants trained with GOLIAH improved time to perform the task in most JA games and imitation scores in most imitation games. GOLIAH intervention did not affect Parental Stress Index scores. At end-point, we found in both groups a significant improvement for Autism Diagnostic Observation Schedule scores, Vineland socialization score, Parental Stress Index total score, and Child Behavior Checklist internalizing, externalizing and total problems. However, we found no significant change for by time × group interaction.

**Conclusions:**

Despite the lack of superiority of TAU + GOLIAH versus TAU, the results are interesting both in terms of changes by using the gaming platform and lack of parental stress increase. A large randomized controlled trial with younger participants (who are the core target of ESDM model) is now discussed. This should be facilitated by computing GOLIAH for a web platform.

*Trial registration* Clinicaltrials.gov NCT02560415

## Background

Autism Spectrum Disorder (ASD) is characterized by the presence of atypical social communicative interaction and behaviours. Typically, ASD is diagnosed by means of behavioural analysis in the 3–5-year age range, and once diagnosed the treatment is mainly delivered through behavioural intervention following different models. In essence, these models try to promote cognitive and behavioural skills that are considered essential for improving social skills and communication in the long run [1–4]. One such program is the Early Start Denver Model (ESDM) protocol, an early and intensive intervention approach for young children with ASD. This program aims to meet the social, developmental and emotional needs of ASD children and their families, and to identify and use validated and effective intervention techniques [5]. The ESDM recently received robust evidence of its efficacy at the level of clinical outcome [1], brain plasticity [6] and a 2-year follow-up [7].

However, two major problems are associated with such interventions. First, given the broad spectrum of ASD with significant inter-child variability, there is a need to design a person specific intervention protocol, accounting for both the actual difficulties/strengths of a child and his/her developmental age, to achieve maximal effects. It has already been established that tailor-made personalized intervention may be more effective compared to any generic type of intervention [8, 9]. Second, at least 20 h/week intensive intervention is needed [10]. The implications of these constraints include the need for trained therapists and the economic cost of such treatments. One way of reducing these problems is to involve parents in the intervention protocol and thereby carry out a significant part of the intervention in home settings. This requires parent training and regular monitoring to check whether the parents properly implement the intervention protocol adhering to that outlined by the therapist.

The use of information communication technologies (ICTs) in therapy offers new perspectives for treating many domains in individuals with ASD because they can be used in many different ways and settings and they are attractive to the patients [11, 12]. Serious games appeared promising because they can support training on many different skills and they favour interactions in diverse contexts and situations, some of which may resemble real life [13]. However, the currently available serious games exhibit some limitations [14]: (1) most of them have limited capabilities and performance in actual interactive conditions; (2) the majority target high-functioning ASD individuals only; (3) their clinical validation has rarely met the evidence-based medicine standards; (4) the game design is not usually described; (5) they have rarely proven their ability of generalization to everyday life. Future research agendas should encompass (1) more robust studies in terms of methodology to assess serious game efficacy; (2) more collaboration between clinical and computer/game design experts; and (3) more serious games that are adapted to young and low-functioning ASD individuals [14].

Since computer based approaches may be effective in improving learning cognitive and social skills in children with ASD [13] and that ESDM received good evidence of its efficacy in young individuals with ASD [1], we settled a multidisciplinary group in the context of the MICHELANGELO European project to fulfil these recommendations, and we recently developed a computerised gaming library (GOLIAH—Gaming Open Library for Intervention for Autism at Home) which consists of a set of computer games created by mapping the imitation and joint attention (JA) subset of stimuli from the ESDM [15]. Imitation and JA are considered to be “pivotal” for the development of communication and social skills which represent core deficits in ASD [15–18]. In GOLIAH, we specifically mapped a subset of ESDM stimuli [1] related to Imitation and JA onto a flexible computer game library containing a set of games (N = 11: 7 related to imitation, 4 related to JA) with varying levels of difficulties that could be reconfigured dynamically by the parent under the supervision of the therapist [14]. In sum, theoretically GOLIAH allows: (1) delivering intervention at home for Imitation and JA tasks in children with ASD; (2) tailoring and adapting intervention through child-specific characterization of difficulties; and (3) allowing dynamic guidance of parental implication.

We tested GOLIAH during a 6-month matched controlled exploratory study. Our aims were to assess (1) the usefulness and acceptability of the gaming platform at home and whether or not the use of relatively intensive parental at home intervention increased parental stress; (2) how experimental children performed using the different Imitation and JA games; and (3) whether children from the experimental group improved significantly more than children treated as usual (control group).

## Methods

### Participants

All children were recruited in the Department of Child and Adolescent Psychiatry, University Hospital Pitié-Salpêtrière, Paris, France and the Department of Child Neuro-Psychiatry, Fondazione Stella Maris, Calambrone, Italy. The study was approved by the local ethics committee of each site (*Comité de Protection des Personnes d’Ile de France VI du Groupe Hospitalier Pitié*-*Salpétrière* under agreement number CCP 21-14 and *Comitato Etico della Fondazione Stella Maris*-*IRCCS* under agreement number 05/2011) was in accordance with the declaration of Helsinki. Each parent (and child when possible) gave informed written consent before inclusion. Inclusion criteria were: a current diagnosis of ASD confirmed by clinical assessment and the Autism Diagnostic Interview-Revised (ADI-R) [19]; an intellectual quotient ≥60; being aged between 5 and 8 years. We excluded children with known organic syndrome and/or non-stabilized neuropediatric (e.g. seizures) or medical (e.g. diabetes mellitus) comorbidities.

We did not randomized patients as the current study was exploratory. We needed to focus on feasibility given the numerus computing requirements of the protocol (wifi EEG at home, transfer from home to hospital of game data, see http://www.michelangelo-project.eu) besides training with GOLIAH. Therefore, inclusion in the experimental group was based on parents’ motivation to follow such a heavy protocol both at home and for the one session per week at the hospital (see below). Controls were matched for sex, age, IQ, study sites and treatment. Treatment as usual (TAU) was defined as all therapeutic interventions given to a specific child. Given the heterogeneity of both severity and needs in ASD individuals, we distinguished two types of TAU for matching based on severity of the cases: first, the cases receiving treatment as outpatients (including speech therapy, occupational therapy, cognitive behavioural therapy/developmental/play therapy, group therapy) with educational support at regular school; second, those receiving day care hospital treatment because associated behavioural problems or autism severity did not permit regular school inclusion. In total, we included 14 children with ASD exposed to GOLIAH (GOLIAH + TAU experimental group) and 10 children with ASD treated as usual (TAU control group). Participants’ characteristics are summarized in Table 1. The contribution of the French and Italian study sites was similar (N = 12 patients, 7 in the experimental group and 5 in the control group).

**Table 1:** Socio-demographic and clinical characteristics of the participants

	Experimental group (N = 14)	Control group (N = 10)
	GOLIAH + TAU	TAU
Demographics		
Age, mean (±SD)	6.85 (±1.34)	7.17 (±1.62)
Male–Female	14–0	10–0
Diagnosis	Autism: N = 3	Autism: N = 3
	ASD: N = 9	ASD: N = 6
	Asperger: N = 2	Asperger: N = 1
ADI-R, current, mean (±SD)		
Social impairment score	14.14 (±4.58)	12.3 (±4.99)
Communication score	10 (±5.82)	8.6 (±4.5)
Repetitive interest score	4 (±2.91)	3.5 (±2.72)
Development score	3 (±1.36)	2.5 (±1.35)
Treatment as usual	As out patient: N = 12	As out patient: N = 9
	In day care hospital: N = 2	In day care hospital: N = 1
	Mean total hours: 15.3 h	Mean total hours: 16 h
TAU details, mean [range] (hours/week)		
Speech therapy	0.57 [0–2]	0.57 [0–1.5]
Psychotherapy^a^	0.33 [0–1.5]	0.75 [0–1.5]
Occupational therapy	0.3 [0–1]	0.2 [0–0.75]
Special education (out of school)	3.66 [0–25]	0.4 [0–4]
Help at school	10.2 [0–30]	14.7 [0–30]

*ADI-R* Autism Diagnostic Interview-Revised, *TAU* treatment as usual, *GOLIAH* Gaming Open Library for Intervention for Autism at Home

^a^Cognitive Behaviour Therapy or Play therapy or Gestald therapy

### Intervention

The control group received TAU according to each site proposal given that both French and Italian health care systems offer free access to medical and educational services.

The experimental group was exposed to TAU plus 5 sessions per weeks of training with GOLIAH: four 30-min sessions per week were at home with the parents playing with their children; 1 session per week was planned at the hospital (see details below). Given the diversity of the games and the heterogeneity of the children’s profiles and abilities, for a given game the number of sessions dedicated to the game varied. Also, given the levels of difficulty within a game, all of the children had more games to play (all the conditions of the games may not have been exploited). Each child’s plan was tailored on the basis of functional profile and adapted during the 6-month protocol according to a child’s progress in playing the games. The hospital session (approximately 1 h/week) was structured as follows: (a) during the first 15 min parental debriefing and planning the following week’s gaming priorities based on the child’s performance at the present time in the gaming platform; (b) 20 min dedicated to structured one-to-one session focused on imitation and joint attention activities with a therapist; (c) 15 min dedicated to repeating on GOLIAH the games preformed with the parents during the preceding week. On average per week each participant was expected to play GOLIAH for 2 h with his/her parents and 15-min with the therapist in addition to the 20-min face to face structure session at hospital.

To tailor treatment given at home therapists had the opportunity to consult the game parameters via a graphic interface that had been implemented in a specific component of Decision Support System (DSS), the Clinical User Interface. It provided a visual feedback on the tasks by highlighting summary performance of the child overtime. This feedback was particularly useful to have access to the child’s results for the sessions conducted at home. This interface assisted the clinician in understanding evolution, compliance and effectiveness of GOLIAH intervention through a very usable Interface with options for comparison of sessions. Thus, clinicians could monitor a child’s progress or difficulties with each game in GOLIAH and adapt the therapeutic intervention for the home-based treatment [15].

### Brief description of the GOLIAH platform

The GOLIAH platform^1^ has been described in details in Bono et al. [15] and offers a series of 11 serious games to stimulate and improve imitation and JA. Serious games can be described as digital/computer games and equipment that provide an agenda of educational design and are beyond entertainment [14]. The multi-player gaming platform developed requires two computers—either tablets or desktop/laptop—that communicate in real time through a multi-threading process. They are connected remotely allowing them to operate from two remote locations. One computer is operated by the therapist or parent (depending upon the application scenario) acting as the *therapist/parent* and the other by the child designated as the *player*. The choice of goal setting as well as the game to play is made by the therapist/parent according to the desired stimuli (JA or Imitation). The role of the player is to achieve the goal set by the therapist/parent at the end of the game. One category of the games is of stand-alone operation, where the therapist/parent needs to select an appropriate game from the pre-developed library and the player is required to execute the game following automated instructions embedded within the game. In the other category, the therapist/parent has an active role to play where he/she needs to cooperate with the child to achieve the goal of the game and has also the flexibility to create new stimuli. All the games have different levels of difficulty allowing the therapist/parent to adjust the initial level of difficulty according to the cognitive skills characterized by the therapist at the beginning of the treatment process or dynamically adjusting it as the player’s performance progresses with time.

The GOLIAH platform selected two important stimuli from ESDM protocol: Imitation and JA. The stimuli were mapped into 11 games, seven for Imitation and four for JA, that were developed by a multidisciplinary team including engineers and clinicians trained in ESDM. The list of the games and the ESDM stimuli they address are depicted in Table 2 and detailed in Bono et al. [15]. In developing the games, special attention has been devoted to their realistic resemblance to the real-life scenario, more importantly emulating human–human interactions during the game playing phase. Each of the games incorporates different levels of difficulty ranging from the application of one stimulus to a combination of different stimuli.

**Table 2:** Mapping of ESDM stimuli for JA and imitation into GOLIAH games

Game type	Description	ESDM stimuli	N of sessions per child: mean [range]
*Imitation games*			
Imitate free drawing	Imitation of the drawing done by the online therapist/parent	(lev.4) FM 4	38 [0–118]
Imitate step by step drawing	Imitation of a drawing created step by step from the online therapist/parent (three difficulties)	(lev.4) FM 4	14.2 [0–43]
Imitate speech	Imitation of words or phrases from the library (three difficulties)	(lev.2) IM 3, 9	22.5 [0–58]
Imitate sounds	Imitation of sounds chosen from the library (four difficulties and two categories of stimuli)	(lev.2) IM 2	29.5 [0–100]
Imitate actions	Imitation of the actions with balls made by the online therapist/parent (three difficulties and two types of task)	(lev.2) IM 6	16 [0–35]
Imitate actions and build	Imitation of the actions with cubes made by the online therapist/parent (three difficulties and two types of task)	(lev.3) FM 3	10.7 [0–28]
Guess the instrument	Identification of the musical instruments played and chosen by the therapist/parent from the library (two difficulties)	(lev.1, 2) IM	9.2 [0–22]
*Joint attention games*			
Follow the therapist’s pointing	Identification of the object indicated (verbally, visually or pointed) by the therapist on the video and chosen from the library (six difficulties and eight categories of stimuli)	(lev.1) RC 1, 4 (lev.2) JA 2, 4, 6	32 [0–109]
Cooperative drawing—connect dots	The therapist and the child cooperate to complete a figure shown on the right, by clicking on the corners of the figure itself (two difficulties and four categories of stimuli)	JA	48.6 [1–124]
Bake a recipe	The child cooks a recipe by clicking and dragging into a bowl the ingredients chosen by the therapist/parent from the library of recipes (11 categories of stimuli)	JA	13 [1–16]
Receptive communication	The child identifies the objects described by the therapist/parent and chosen from the library (three difficulties and five categories of stimuli)	(lev.2) RC 5, (lev.1) RC 6, (lev.1) RC 4	53.4 [4–112]

*FM* fine motor subset, *IM* imitation subset, *RC* receptive communication subset, *JA* joint attention subset

The gaming platform provides a flexible means for giving a reward to the player on successful completion of the goal capturing the essence of reward-based intervention. A smiley face is shown at the end of each game in the player’s device, regardless of the score obtained as a positive reinforcement which also gives an impression of feedback to the player. Such feedback is once again programmable, and an appropriate reward could be set by the therapist depending on the player’s motivation factors (such as playing music that the child likes, etc.).

### Automatic extraction of parameters from the serious game

The performance of the player while playing the game was assessed mainly in two different ways: (1) automated evaluation based on a predefined scoring convention and (2) manual evaluation by the therapist/parent. A scoring system of 0–2 has been implemented for this purpose where 0 means the player did not achieve the goal, 1 for partial achievement and 2 for successfully satisfying the goal. Apart from the simple scores describing whether the player has achieved the goal, a set of objective metrics and an array of possible events are also extracted by the platform in an automated way. This set of objective metrics allows the therapist to analyse quantitatively the performance of the player in a stimulus-specific way not only at a particular time point but also during the progression of the child’s performance over a time window (hours, days, months, etc.) giving a holistic picture of the child’s development. In addition, this also allows the therapist to ascertain the appropriateness of scoring and adherence to the prescribed protocol by the parents. Such analysis could be done both online and offline by the therapist as the metrics are stored each time the player plays the game.

From the experimental group exposed to GOLIAH, several parameters were saved more or less automatically (depending on the games) from the different games implemented in the tablet serious game. (1) Date and time, task (imitation or JA), game number, level number; (2) The reaction time (RT) that corresponds globally to the time used by the child to complete a task. (3) Scores that correspond to wrong or correct answers (automated evaluation) and good or bad completion (therapist’s evaluations: failed, partially achieved, or well done) of the task.

### Clinical measures

To assess clinical change during the 6-month exploratory study, using a single blind procedure we measured the following variables at enrolment and at 6-month outcome. Double blind was not possible given parents’ participation in the GOLIAH protocol. The primary outcome variable was the *Autism Diagnosis Observation Schedule* (ADOS) which is a tool for autism diagnosis. We used the communication and social interaction scores, and the Communication + Interaction score (later called ADOS total score) [20]. Secondary variables included: (1) the *Vineland Adaptive Behavior Scale II* (VABS-II) [21] as a behavioral scale of independence which is a parent interview used to assess the ability of children to perform the daily activities required for personal and social sufficiency. The VABS-II examines four specific domains: Communication, Daily Living Skills, Socialization, and Motor Skills. The subscale scores are added up to yield an Adaptive Behavior Composite score. (2) *Wechsler scales,* a standardized developmental test for children to measure Intelligence skills (WPPSI III & WISC IV) [22, 23], which offer Verbal, Performance, Working memory, Processing Speed and Total quotients. (3) The *Child Behavior Checklist* (CBCL) to assess global psychopathology [24]. It is a 100-item parent-report measure designed to record the behaviors of preschoolers. Each item describes a specific behavior and the parent is asked to rate its frequency on a three-point Likert scale. The scoring gives, among others, three main scores (Internalizing, Externalizing, Total Problems): a T-score of 63 and above is considered clinically significant; values between 60 and 63 identify a borderline clinical range; values under 60 are considered not-clinical. (4) The *Social Communication Questionnaire* (SCQ) to assess communication more specifically [25]. It is completed by parents and evaluates communication skills and social functioning of children. SCQ provides a Total Score that can be interpreted in relation to specific cut-off points (over 15 is considered indicative of a risk for ASD). SCQ content parallels that of the ADI-R, and the agreement between the two instruments is high and substantially unaffected by age, gender, language and performance IQ. (5) The *Parenting Stress Index (PSI),* to assess parental stress during the study [26], is designed to evaluate the magnitude of stress in the parent–child system. The scoring gives a Parent Domain score (including the sum of the raw scores of the following subscale: Competence, Isolation, Attachment, Health, Role Restriction, Depression, and Spouse), a Child Domain score (including the sum of the raw scores at following subscale: Distractibility, Adaptability, Reinforces Parent, Demandingness, Mood, and Acceptability) and a Total Stress score that is the sum of Parent and Child Domain raw scores (higher raw scores both at PSI Scales and subscales mean more parent stress).

### Statistical analysis

Given the exploratory nature of the study, there was no assumption of the sample size. Besides this limitation, we performed statistical analyses using R Software (Version 2.12.2). To assess whether adding GOLIAH relatively intensive exposure to TAU improved both primary and secondary clinical variables, we used Linear Mixed models with change in the given variable to be explained by group exposure (TAU vs. TAU + GOLIAH), time (baseline vs. 6-month) and their interaction (group exposure × time). We also included a random effect for participants and a site effect. This allows taking into account individual heterogeneity, site heterogeneity, variable scores at inclusion and change specific to exposure to GOLIAH within the same statistical regression. In the experimental group, in order to assess whether children improved we focused on the reaction time for JA games and the imitation scores (failed, intermediate, or well done) for imitation games. In the case of “bake a recipe” game, we explored the time to complete the task (TCT) as this game is a multistep complex task. We used Linear Mixed Models (or Ordinal Mixed Model) with change in the reaction time (or change in the imitation score) to be explained by time (or consecutive sessions), difficulty levels and/or eventually the number of items (see Table 3). In case of non-normal distribution, we studied variable log transformation to reach normal distribution.

**Table 3:** Performance changes of GOLIAH trained children through sessions for all JA and imitation games

Game type (variable, n = events)	N	Time effect	Difficulty level effect	Number of items effect
Imitation games				
Imitate free drawing (Imitation score per drawing—n = 562)	13	Imitation score increases with session (β = 0.02, p = 0.036)*	NA	NA
Imitate step by step drawing (Imitation score per drawing—n = 198)	13	No effect (β = 0.13, p = 0.089)*	NA	Imitation score increases with the number of steps (β = 0.27, p = 0.047)*
Imitate speech (Imitation score per words or sentences—n = 315)	13	No effect (β = 0.014, p = 0.61)*	No effect (β = −0.21, p = 0.328)*	No effect (β = −0.029, p = 0.72)*
Imitate sounds (Imitation score per sounds—n = 452)	13	Imitation score increases with session (β = 0.037, p = 0.019)*	Imitation score decreases when increasing severity (β = −0.31, p = 0.014)*	No effect (β = 0.05, p = 0.41)*
Imitate actions (Imitation score per actions—n = 161)	13	Imitation score increases with session (β = 0.11, p = 0.039)*	No effect (β = 0.089, p = 0.86)*	No effect (β = −0.43, p = 0.18)*
Imitate actions and build (Imitation score per contruction—n = 227)	13	Imitation score increases with session (β = 0.149, p < 0.001)*	No effect (β = 0.266, p = 0.47)*	Imitation score decreases with the number of cubes (β = −0.1′, p = 0.0176)*
Guess the instrument	13	Bug	Bug	Bug
Joint attention games				
Follow the therapist’s pointing (RT for good answers—n = 681)	13	RT decreases with sessions (β = −0.0045, p = 0.048)**	No effect (β = −0.014, p = 0.247)**	No effect (β = 0.0057, p = 0.428)**
Cooperative drawing—connect dots (RT—n = 449)	13	RT decreases with sessions (β = −0.024, p = 0.045)**	NA	No effect (β = 0.0035, p = 0.51)**
Bake a recipe (TCT—n = 748)	14	RT decreases with sessions (β = −0.021, p < 0.001)**	NA	NA
Receptive communication (RT for good answers—n = 225)	14	No effect (β = −0.002, p = 0.776)**	RT is faster in easy versus difficult condition ( = −0.17, p = 0.021)**	NA

*N* number of children exposed to the game during at least 2 sessions (as opposed to n = events that corresponds to the number of tasks with a given score included in the statistical regression), *RT* reaction time (to perform the task); *TCT* time to complete the task, *NA* not appropriate

* Ordinal mixed models; ** Linear mixed models with log transformation

## Results

### Acceptability and parental stress

Given the study design, a 6-month treatment meant at maximum 100 sessions (4 sessions with parents at home per week + 1 session with a therapist at the hospital per week = 5 sessions per week × 20 weeks = 100 sessions, taking into account a 4-week summer vacation during the study period). Overall, there was no study dropout. However, three children had fewer than 12 sessions. Children and parents participated in 30.5% of the planned sessions at home and in 48.6% of the hospital sessions, which led to a total participation of 39.9%. When excluding 3 children showing poor participation, we found that 38% of the sessions at home and 61.8% of the hospital sessions were provided. This means that the participation of the parents at home made children’s exposure to GOLIAH to be multiplied by a factor 2.66 compared to exposure only during sessions with a therapist. Given the diversity of the games and the heterogeneity of children profile and abilities, for a given game the number of sessions dedicated to that game varied. Also, given the levels of difficulty, within a game, all of the children had more games to play (not all of the conditions of the games have been exploited). However, all games were used during the study period (see right column of Table 2) with *guess the instrument* being the least played (mean number of sessions per child = 9.2 [range 0–22]) and *receptive communication* being the most played (mean number of sessions per child = 53.4 [range 4–112]). All games were well tolerated and followed both by children and parents showing the robustness of the gaming platform and the feasibility of the course of the games. One family initially had trouble using the two tablets system related to Wi-Fi connecting problems that were easily corrected. Tailoring treatment during the hospital session and data transfer from home was also easily achieved.

To assess the magnitude of stress in the parent–child system during the protocol, we used the *Parenting Stress Index (PSI).* To compare course of stress at 6 months, we used Linear Mixed model with two main effects: group (Experimental vs. Control) and time (Inclusion vs. 6 months). This allows taking into account individual heterogeneity, variable scores at inclusion and change specific to exposure to GOLIAH within the same statistical regression. Results are shown in Tables 3 and 4. There was a significant improvement at 6 months in both groups for PSI parental distress, difficult child, and total stress scores (all *p* < 0.05); meaning that treatment given in both groups was positive in terms of stress for almost all variables. However, there was only a statistical tendency at 6-months for improvement of dysfunctional interaction (p = 0.065). Interestingly, we found no significant effect of groups, meaning that being included in the experimental group and for the parent being directly and intensively involved in therapeutic sessions did not increase parental stress.

**Table 4:** Clinical variables of the participants and Parental Stress Index at baseline and 6-month outcome

	T0 = Baseline		T6 = Outcome at 6 months	
	Experimental group (N = 14)	Control group (N = 10)	Experimental group (N = 14)	Control group (N = 10)
ADOS, mean (±SD)				
Communication score	3.6 (±1.7)	4.5 (±1.5)	2.9 (±2.1)	3.7 (±1.6)
Interaction score	7.2 (±2.4)	9.3 (±2.6)	6.3 (±2.8)	7.3 (±2.9)
Total score	10.8 (±3.6)	13.8 (±3.5)	9.2 (±4.6)	11 (±4.1)
Cognition WISC3/WPPSI				
VIQ	103.1 (±14)	100.8 (±25.9)	107.2 (±22.8)	102.4 (±26.6)
PIQ	96.1 (±24.8)	96.4 (±24.5)	104.4 (±19.9)	98.1 (±24.5)
Speed	93.5 (±12.6)	90.6 (±16.2)	97.4 (±15.3)	90.7 (±21.9)
Working memory	107.6 (±21.5)	97.8 (±28.2)	107.4 (±23.6)	99.2 (±27.6)
Total IQ	98.8 (±20.1)	96.3 (±22.7)	107 (±20.8)	98.9 (±25.8)
Vineland, mean (±SD)				
Communication score	88.2 (±16.7)	86.2 (±13.9)	79.6 (±11.5)	82.8 (±6.5)
Daily living	84.3 (±13.4)	85.4 (±14.7)	79.4 (±5.5)	83.6 (±10.8)
Socialization	79.5 (±10.3)	80.1 (±11.9)	78.3 (±10.7)	85.3 (±8.4)
SCQ, mean (±SD)				
Total score	11.6 (±7.7)	11.5 (±7.2)	10 (±6.3)	8.6 (±7)
CBCL T score, mean (±SD)				
Withdrawn/depressed	62.8 (±9.9)	62.9 (±9.5)	60.9 (±8.6)	60.6 (±9)
Somatic complaints	56 (±7.7)	59.5 (±7.5)	54.7 (±7.6)	57.5 (±8.3)
Anxious/depressed	62.8 (±8.1)	61.2 (±9.3)	60.5 (±7.6)	58.6 (±10.5)
Social problems	68.5 (±6.5)	66.7 (±7.4)	63.8 (±7.3)	60.8 (±8.2)
Thought problems	61.1 (±10.8)	66.7 (±8.4)	59.8 (±10.8)	61.2 (±9.5)
Attention problems	65.1 (±9.1)	67.4 (±9.1)	63.6 (±9.7)	61.1 (±9.7)
Rule-breaking behavior	58.6 (±7.4)	58.1 (±6.1)	56.5 (±6.3)	57.4 (±6.2)
Aggressive behavior	60.4 (±6.5)	64.4 (±9.2)	57.8 (±6)	61 (±8.5)
Internalizing	62.5 (±9)	63 (±8)	60.2 (±9.5)	59 (±10.3)
Externalizing	59.1 (±8.1)	61.7 (±7.1)	57.7 (±7.7)	55.9 (±11.8)
Total	63.9 (±8.4)	66.5 (±7.3)	61.2 (±8.3)	60.5 (±11.3)
PSI, mean (±SD)				
Parental distress	31.6 (±6.9)	32.9 (±8.2)	28.3 (±9.4)	27.6 (±6.5)
Dysfunctional interaction	31.5 (±5.7)	37.3 (±6.4)	26.8 (±8.8)	28.3 (±7.4)
Difficult child	37.2 (±7.3)	39.2 (±7.2)	29.4 (±7.3)	30.3 (±8.2)
Total stress	100.7 (±15)	105.8 (±13.6)	85.5 (±22.6)	86.2 (±20)

*ADOS* Autism Diagnostic Observation Schedule, *WISC 3* Wechsler Intelligence Scale for Children 3, *WPPSI* Wechsler Preschool and Primary Scale of Intelligence, *VIQ* Verbal Intelligent Quotient, *PIQ* Performance Intelligent Quotient, *CBCL* Child Behaviour Checklist, *PSI* Parental Stress Index

### Children’s performance across sessions and games in the experimental group

Changes of children’s performances across sessions for all Imitation and JA games are shown in Table 3. All analyses were multivariate with repeated measures modelled with a random effect for participants (to control for individual variation) and a site effect (to control possible biases between Paris and Pisa sites). We distinguished time effect and eventually difficulty levels within the task, and the number of items. Unfortunately, we could not perform statistical analysis for data from the “Guess the instrument” imitation game due to a computational bug when storing the data. We found a significant improvement of the imitation score (corresponding to well done completion of the imitation task score) in 4 among the 6 remaining imitation games (“Imitate a free drawing”, “Imitate sounds”, “Imitate actions”, and “Imitate actions and build”). Since we used log transform and multivariate models, β coefficients are not immediately understandable for their clinical relevance. Within Ordinal Mixed Models, we modelled a log (odds ratio). Thus, by exponentiating the beta we obtained the increase (or decrease) in imitation score. Contrary to a binary logistic regression, the dependent variable has more than two categories. In our case, we have 3 modalities: failed, partially achieved, and well done. The interpretation is quite similar to a binary logistic regression, except that a category is compared to the combined greater (or lower) categories. The following example should help reading Table 3 for imitation games. For “Imitate free drawing”, we found a significant effect by time and β was equal to 0.02 (p = 0.036). In other words, after 10 sessions of training, the score increased by a factor equal to e^10 × 0.02^ = 1.22 = 1 + 0.22 which means that a child who failed increases of 22% his/her chances to (partially) achieve the game after 10 sessions of training.

Also, we found a significant improvement of the time to perform the task in 3 among 4 JA games (“Follow the therapist’s pointing”, “Cooperative drawing Imitate sounds”, “Bake a recipe”). As explained above, β coefficients are not immediately understandable for their clinical relevance. Within Linear Mixed Models, we modeled the log (Reaction Time). Thus, by exponentiating the beta we obtained the increase (or decrease) in Reaction Time. Taking “Follow the therapist’s pointing” as an example, we found a significant effect by time and β was equal to −0.0045 (p = 0.048). This means that children decreased significantly their JA reaction time by 5% every 10 sessions of training (e^10 × (−0.0045)^ = 0.95, so after 10 sessions the reaction time represents 95% of the initial reaction time). We conclude that participants improved their abilities to perform most of the imitation and JA games during relatively intensive training with GOLIAH.

### Improvement of clinical measures in the experimental versus control groups

Table 4 summarizes all participants’ clinical measures and PSI scores at baseline and at 6-month outcome for both groups, under experimental treatment (TAU + GOLIAH) or under control condition (TAU). Clinical variables included ADOS communication, interaction and total scores, Vineland communication, daily living and socialization scores, Wechsler cognitive scores, SCQ score and CBCL 8-subscale scores and CBCL internalizing, externalizing and total scores.

To assess improvement at 6 months we used Linear Mixed models with two main effects: group (Experimental vs. Control) and time (Baseline vs. 6 months). Results are shown in Table 5. At end-point, we found no significant change for by time × group interaction. Also, we found no significant group effect for all variables (all p > 0.05, Linear Mixed Models); meaning that the GOLIAH platform given in a relatively intensive way at home and hospital failed to show a generalization of its effect in improving social (e.g. Vineland), cognitive (e.g. IQ) or core symptoms (e.g. ADOS) of ASD. However, we found a significant time effect. There was a significant improvement for Autism Diagnostic Observation Schedule (ADOS) scores, Vineland socialization score, Parental Stress Index total score, and Child Behavior Checklist internalizing, externalizing and total problems (all p < 0.05, Linear Mixed Models, time effect); meaning that treatment given in both groups was positive. There was only a statistical tendency for Social Communication Questionnaire score (p = 0.054).

**Table 5:** Change in clinical variables at 6 months (linear mixed models)

	Group effect	Time effect	Group × time interaction
ADOS, mean (±SD)			
Communication score	−0.93 (p = 0.22)	−0.8 (p = 0.016)	0.16 (p = 0.7)
Interaction score	−2.07 (p = 0.071)	−2 (p = 0.008)	1.07 (p = 0.25)
Total score	−3.01 (p = 0.082)	−2.8 (p = 0.001)	1.23 (p = 0.21)
Cognitive level (WISC3/WPPSI)			
VIQ	2.3 (p = 0.8)	1.7 (p = 0.63)	2.5 (p = 0.59)
PIQ	−0.33 (p = 0.97)	1.7 (p = 0.64)	6.66 (p = 0.16)
Speed	2.99 (p = 0.69)	0.11 (p = 0.98)	3.7 (p = 0.53)
Working memory	9.85 (p = 0.42)	2.14 (p = 0.49)	2.87 (p = 0.56)
Total IQ	2.5 (p = 0.8)	2.55 (p = 0.46)	5.61 (p = 0.23)
SCQ, mean (±SD)			
Total score	0.071 (p = 0.98)	−2.9 (p = 0.054)	1.33 (p = 0.49)
Vineland: mean (±SD)			
Communication score	8.6 (p = 0.14)	3.2 (p = 0.27)	−5.2 (p = 0.17)
Daily living	4.89 (p = 0.34)	4.2 (p = 0.19)	−3.06 (p = 0.46)
Socialization	1.2 (p = 0.79)	7 (p = 0.033)	−6.29 (p = 0.13)
CBCL T score: mean (±SD)			
Withdrawn/depressed	−0.54 (p = 0.99)	−2.3 (p = 0.22)	0.38 (p = 0.88)
Somatic complaints	−3.5 (p = 0.3)	−2 (p = 0.2)	0.69 (p = 0.73)
Anxious/depressed	1.57 (p = 0.68)	−2.6 (p = 0.1)	0.37 (p = 0.86)
Social problems	1.83 (p = 0.58)	−5.89 (p = 0.04)	1.14 (p = 0.64)
Thought problems	−5.58 (p = 0.23)	−5.44 (p = 0.12)	4.11 (p = 0.37)
Attention problems	2.32 (p = 0.56)	−6.3 (p = 0.011)	4.84 (p = 0.12)
Rule-breaking behavior	0.47 (p = 0.87)	−0.67 (p = 0.64)	−1.42 (p = 0.46)
Aggressive behavior	−4.46 (p = 0.18)	−3.44 (p = 0.054)	1.23 (p = 0.59)
Internalizing	−0.54 (p = 0.89)	−4. (p = 0.018)	1.77 (p = 0.4)
Externalizing	−2.62 (p = 0.48)	−5.8 (p = 0.01)	4.41 (p = 0.12)
Total	−2.58 (p = 0.5)	−6 (p = 0.013)	3.2 (p = 0.28)
PSI, mean (±SD)			
Parental distress	−1.51 (p = 0.65)	−5.55 (p = 0.037)	2.19 (p = 0.5)
Dysfunctional interaction	−2.13 (p = 0.5)	−5.68 (p = 0.065)	0.61 (p = 0.87)
Difficult child	−2.67 (p = 0.4)	−9.59 (p < 0.001)	1.8 (p = 0.41)
Total stress	−6.4 (p = 0.42)	−20.92 (p = 0.002)	5.7 (p = 0.47)

*ADOS* Autism Diagnostic Observation Schedule, *WISC 3* Wechsler Intelligence Scale for Children 3, *WPPSI* Wechsler Preschool and Primary Scale of Intelligence, *VIQ* Verbal Intelligent Quotient, *PIQ* Performance Intelligent Quotient, *CBCL* Child Behaviour Checklist

## Discussion

### Summary of the results

Here we report the results of a 6-month controlled trial testing the use of GOLIAH as a relatively intensive adjunct treatment provided at home by the parents through 30-min sessions and under weekly supervision at hospital. We included 14 children with ASD in the experimental group, and 10 controls matched for diagnosis, gender, age, sites, and TAU. Despite the extensive parental contribution in the experimental group, GOLIAH intervention did not affect Parental Stress Index scores. There was a significant improvement of PSI scores in both groups. This means that participation in the experimental group did not increase parental stress. All games were well tolerated and followed both by children and parents showing the robustness of the gaming platform and the feasibility of the course of the games. Therapists could easily tailor treatment during the hospital session based on data transferred from home. We found a significant improvement in 4 among 6 imitation games on the quality imitation scores and 3 among 4 JA games on the time to complete the task across sessions. This confirms that training participants with ASD using computer based approaches may be helpful (e.g. Serret et al. [27]) although this does not imply that participants may generalize their improved abilities outside the gaming context [14]. In terms of feasibility, we need to specifically discuss acceptance. On one hand, acceptance of GOLIAH was good since we had no drop out during the study. On the other hand, regarding intensive exposure to GOLIAH, the overall observance rate of nearly 1 session done for 2 predicted sessions is disappointing. It shows that implicating parents may be more complex than expected despite declared motivation. To improve acceptance in the future, we propose that at home family intervention should be supported by the development of an app to recall using GOLIAH and to facilitate real-time assessment of the child in his/her natural environment (Ecological Momentary Assessment).

However, the primary outcome of the trial was negative. At end-point, we found no significant change for by time × group interaction. We found a significant improvement in both groups (i.e., trained or not with GOILAH) for ADOS scores, Vineland socialization score, Parental Stress Index total score, and Child Behavior Checklist internalizing, externalizing and total problems. The lack of significant group effect means that the GOLIAH platform given in a relatively intensive way at home and hospital failed to show a generalization of its effect in improving social (e.g. Vineland), cognitive (e.g. IQ) or core symptoms (e.g. ADOS) of ASD. Other possible explanations for improved performance over 6 months in both groups may be children’s maturation, test–retest advantage and un-blinded parents providing the ratings for VABS, CBCL, PSI questionnaires. The lack of adverse outcome (e.g. increase of parental stress) allows us to plan a larger randomized controlled trial given the sample size of the current exploratory study. To address the negative results on our primary variable, we will discuss protocol changes. Given that (1) the ESDM protocol has been implemented for children younger than 5 years [1] and that (2) several authors have highlighted the better outcome when treatment of children with ASD starts earlier in age [4], we are planning to focus on younger children. Also, in its current status, GOLIAH platform is not implemented in a web site, but a web version may be easier for parents to observe and may help to limit the number of sessions at hospital and to increase treatment participation at home. We are now computing a web version of GOLIAH to be made available on Curapy (www.curapy.com/) a web platform for e-health serious games.

### GOLIAH compared to other serious games in ASD

It is not under the scope of this manuscript to review information communication technologies (ICTs) commonly used in autism assessment and therapy. From existing reviews [13, 14, 28–30], we maintain that serious games are particularly promising because of their many treatment possibilities and their attractiveness for participants. In the next paragraphs, we wish to highlight GOLIAH’s original characteristics in comparison with already existing serious games.

#### Targeted skills and population

The first originality of GOLIAH platform concerns the choice of the targeted skills—imitation and joint attention—as part of the premises for social learning. Only a few of the serious games are related to these skills and sometimes they are not their first target as the majority of serious games aim to foster higher-level skills such as communicational or emotional skills. For instance, CopyMe [31] requires one to recognize an emotion from a picture and mimic that expression; FaceSay is intended to improve joint attention skills [32]. But the main focus of these two games concern, more generally, emotion and facial recognition. Among games oriented towards communicational skills, some of them require the child to create joint attentional interactions with an avatar [33, 34] or with a partner [35, 36]. However, none of them directly address the measuring of imitation/joint attention such as GOLIAH does and, as far as we know, no other game is designed to train conjointly these two precursors of communication.

In terms of population, the easiness of GOLIAH games, as well as their intention to target low-level skills, make this platform accessible to younger children and children with a severe degree of autism, which is relatively uncommon among existing games. The majority of games target older children or adolescents with ASD, and many of them are intended for people with HF-ASD [28, 29]. Only a few other games are specifically meant for younger or LF-ASD children [27, 35, 37–39].

#### Clinical inspiration

Effective treatment in ASD such as ABA, TEACCH, ESDM are challenging to implement and require intense exposition [4]. Serious games represent a potential alternative to apply these approaches (easy access; low-cost; possible at home therapy). Also, they provide storable and accessible data on which clinicians can rely to evaluate the children’s progress. Unfortunately, very few games are based on these principles [40]: ComFim is inspired by PECS [35], Invasion of the wrong planet relies on TEACCH [41], TeachTown incorporates the basic principles of ABA [39]. GOLIAH is the first to be based on ESDM. In addition, not only are the different games of GOLIAH inspired by ESDM tasks but they also respect some general principles of the program: developmental perspective (levels of difficulty that the child has to achieve to progress through the game); clinicians can assess the progress of the child and review objectives by adapting the choice of games; a wide variety of games (11 games) which guarantees obtaining an intensive, adjustable enrichment therapy tailored for each child. The diversity and incremental perspective of GOLIAH games are real assets for therapy, in comparison to serious games standing in one only game which tend to be repetitive, not challenging enough or simply not attractive for the children [40] (e.g. CopyMe [42], SmileMaze [43] or SIDES [44]).

#### Choice of the media and gameplay

The GOLIAH gaming platform has been specifically designed to be played on two connected digital tablets. Tablets have many advantages over more traditional media: convenient and portable format, interface adapted to the children, easy use and affordable price. As digital touch device, it allows a richer interactivity (e.g. direct manipulation of digital objects on the surface) and does not require hand-eye coordination capabilities as is the case when using a computer mouse. Finally, through its very playful aspect the tablet seems to generate an increased motivation and could also promote interactions between subjects in an intuitive and natural way [43–46].

A lot of existing serious games rely on avatar technology [33, 34, 47] which seems really efficient with ASD people who appreciate interactions with virtual agents because of their predictable behaviors [48, 49]. However, the majority of computer games for social skills development are designed for one user working directly with the application and lack the face-to-face interaction found in authentic social situations [11]. Interesting alternatives are provided by applications that support co-located interaction of multiple users, i.e. joint activities that are carried out by two or more people located in the same place [36]. The majority of studies proposing co-located interactions have used touchscreen or tabletop interfaces which have demonstrated their effectiveness for training social skills by involving pairs [36, 38, 50] or small groups [11] of ASD children. To date, the game ComFim [35] is the only other game referring to the simultaneous use of two tablets.

Indeed, the fact of using two digital tablets also represents another innovative aspect of GOLIAH because the game has been used as a media of interaction between children and caregivers. Modern interventions have emphasized the need for caregivers to share the management of goals for each child through co-creation of learning experiences [51]. Teachers and parents look to technology as a complimentary support but there is, unfortunately, a notable paucity of autism related applications involving caregivers and use at home [34]. In the Junior Detective Training Program [52], home missions requiring parents’ participation are limited to parents helping the children with the completion of ‘Secret Agent Journal’ entries, a portion of the game which allows children to document between-session activities. Similarly, for « Let’s Face it » [53] the role of the parents is to send log files on a weekly basis, as the game has been designed to be played self-paced and not directly supervised by the parent or caregiver.

Also, when therapists are involved during game sessions they usually have more a role of guidance providing feedback to the participants (i.e. [54, 55]). In GOLIAH, in addition to this traditional guidance role, some of the games were built to directly practice joint actions with the therapist or the caregiver in a more playful way. As far as we know, GOLIAH is the only gaming platform that can be used both with clinicians at the hospital and with parents at-home. Yet, according to the feedback of clinicians and parents using GOLIAH, the at-home natural environment and the dialog established between parents and therapists were key factors of the attractiveness of the rehabilitation. In particular, from self-report questionnaires that were administered to parents at 3 months, 66% of families involved in the study thought that there was a specifically attractive aspect related to the media itself [15]. Moreover, the quality of parent–child relationship was qualified as enhanced for 55% of the parents, and some parents have attributed specifically that amelioration to the use of the digital tablet which allowed them to create a playful common space to interact with their child [15].

### Limitations

The main limitation of the study relies on the exploratory nature of the design. First, given the important involvement of the parents, we decided not to randomize experimental treatment attrition and rather selected the most motivated parents to enter the GOLIAH adjunct treatment. As a consequence, we matched individuals for the control group on several variables to limit biases in the outcome comparison. However, this process might have introduced biases. As shown in Tables 4 and 5, there was a statistical tendency for ADOS interaction and total score for a group effect, due to participants selected in the control group that tended to be more severe. However, the nearly 50% observance rate after 6-months shows that we were right to select highly motivated parents. Second, the small sample size may have limited our statistical power and prevented detecting relevant clinical changes with GOLIAH adjunct treatment such as those related to core symptoms of autism (ADOS and SCQ scores). Third, the nearly 50% observance rate was obtained in the context of a research agenda with a numerous and available support team. We wonder whether observance would decrease in a more conventional clinical context. There is a need to offer an easy and friendly web interface for GOLIAH before recruiting for a larger trial. Fourth, we cannot exclude that the “active ingredient” of the GOLIAH treatment package was the weekly in-clinic sessions with a therapist working on the same specific targets rather than the computer game interface. Finally, the last limitation regards how GOLIAH differs from ESDM. Compared to the ESDM protocol to which the GOLIAH platform refers [5], the children in the current study were older and received less intense treatment (in terms of hours, see Table 1) which may have constrained their ability to change with treatment.

## Conclusion

GOLIAH platform combines the affordance of face-to-face interaction with the benefits of computer games as a reassuring, predictable and structured environment [56] in line with ASD population’s preference for consistency and rules [57, 58]. Moreover, it relies on an affordable technology that can be easily used at home with parents and/or by clinicians both to foster interactional social skills and to monitor treatment. The results of the 6-month training are encouraging, both in terms of changes by using the gaming platform and the lack of parental stress increase. However, in the context of this exploratory study, we were unable to show any superiority compared to TAU on core symptoms of autism. Research should now be moved to a large randomized controlled trial with younger participants who are the core target of ESDM model.

## Abbreviations

ABA: applied behavioral analysis
ADI: Autism Diagnostic Interview
ADOS: Autism Diagnostic Observation Schedule
ASD: Autism Spectrum Disorder
CBCL: Child Behavior Checklist
ESDM: Early Start Denver Model
GOLIAH: Gaming Open Library for Intervention in Autism at Home
ICT: information communication technologies
PECS: picture exchange communication system
PSI: Parenting Stress Index
SDC: Social Communication Questionnaire
TAU: treatment as usual
TEACCH: Treatment and Education of Autistic and related Communication Handicapped Children

## Footnotes

1 GOLIAH is available under request at the University of Southampton by mailing Koushik Maharatna (km3@ecs.soton.ac.uk).
